# Effects of Guarana and Green Tea Consumption on Students’ Intellectual Performances

**DOI:** 10.3390/nu17061000

**Published:** 2025-03-12

**Authors:** Valentina Amaritei, Petronela-Elena Buruiana, Roxana Filip, Florin Filip, Ancuta Veronica Lupaescu, Monica Iavorschi, Roxana-Elena Gheorghita

**Affiliations:** 1 Faculty of Medicine and Biological Sciences, Stefan cel Mare University of Suceava, 720229 Suceava, Romania; valentina.amaritei@student.usv.ro (V.A.); petronela.buruiana@student.usv.ro (P.-E.B.); roxana.filip@usm.ro (R.F.); ancuta.lupaescu@usm.ro (A.V.L.); monica.iavorschi@usm.ro (M.I.); roxana.puscaselu@usm.ro (R.-E.G.); 2 Suceava County Emergency Clinical Hospital, 720001 Suceava, Romania

**Keywords:** cognition, healthy, supplements, nutrition

## Abstract

Background: guarana and green tea are known as compounds that may improve cognitive performance due to their high content of caffeine and other neurostimulants, such as theobromine in the case of guarana and ECGC (apigalocatechin-3-galate) in that of green tea. Methods: this study investigates the effects of *Paullinia cupana* (guarana) and *Camellia sinensis* consumption on students’ intellectual performance. The study group consisted of 33 students, mostly women, from various faculties. During the studied period, they consumed 2 g guarana per day for ten days and 1.5 g green tea per day for ten days. The students completed three cognitive tests before and after administration of the products, measuring reaction speed, memory, and attention. They also completed questionnaires regarding health status, product information, product quality, purchase intention, consumer confidence in the product, and perception of effects and preferences. Results and discussion: the results showed that both guarana and green tea fostered significant improvements in cognitive performance. However, more people felt the effect of guarana than the effect of green tea. The study found a strong correlation between cognitive effects and guarana administration, including improvements in energy and retention of information. For green tea, a calming effect and state of well-being were the most relevant responses. Conclusions: the findings suggest that guarana and green tea may constitute a useful strategy for improving academic performance. However, further research is needed to determine optimal doses, frequency of consumption, and potential long-term effects on cognitive function.

## 1. Introduction

Cognition is the mental process of acquiring knowledge and understanding through experience and the senses. Cognitive abilities include perception, memory, language, attention, executive function, psychomotor functions, and information processing, as well as the application of knowledge and the ability to change preferences. Most cognitive-enhancing compounds are nervous system stimulants that can improve physical and mental performance. These products activate the sympathetic nervous system by releasing adrenaline, and by stimulating neurotransmitters such as norepinephrine, dopamine, glutamate, or serotonin in the central nervous system. Nootropics are substances that can enhance cognitive function in healthy individuals by acting as direct or indirect agonists of dopamine receptors, vasodilators, or by increasing glutaminergic neurotransmission [1]. The most important cognitive-enhancing substances found in guarana and green tea are the following:

- Caffeine is a trimethylxanthine, a naturally occurring substance found in coffee, chocolate, guarana, and other herbs such as cassina, kola nuts, and yerba mate. The effect of caffeine on cognitive performance is likely related to its competitive inhibition of adenosine receptors, which can affect perception of pain, fatigue, stress, and cognition [2]. It acts as a stimulant for the heart, respiratory tract, and central nervous system, and also is a vasodilator and diuretic [3].
- Guaranine is a chemical compound found in guarana, and it is identical to caffeine derived from other sources such as coffee, tea, and yerba mate [4]. Guaranine blocks the A1 and A2 receptors of adenosine, which are present in all parts of the brain, particularly in the hippocampus, cerebral cortex, cerebellar cortex, and thalamus. These chemicals increase alertness, reduce fatigue, and can elevate mood. Normal consumption enhances performance in tasks that require vigilance, such as simulated driving tasks [5].
- Theobromine has a similar effect to caffeine on the human nervous system, although it is less potent. Theobromine is an isomer of theophylline and paraxanthine, mainly present in green and black tea, guarana, and cocoa. Some studies have suggested that theobromine may have anticancer properties [6]. In placebo-controlled studies, theobromine had minimal effects on mood and attention, except for enhancing the effects of other drugs [7].
- ECGC (epigalocatechin-3-galate) is a type of catechin, mainly present in green tea. It has been shown to inhibit the production of reactive nitrogen species (RNS), making it a potent and effective neuroprotective treatment for neurological diseases. Consumption of EGCG has been linked to improved cognitive performance, insulin sensitivity, and brain amyloid formation. It also reduces neuroinflammation and prevents neuropathology associated with neurodegenerative disorders [8].

Guarana is a climbing shrub that is native to the Amazon forests. It is known for its antioxidant, antibacterial, and antimicrobial properties, as well as its cytoprotective, anticancer, and fat-burning effects. Guarana powder has been found to offer cognitive benefits, particularly in memory, attention, decision-making speed, and processing/response speed. These cognitive benefits are greater than those of caffeine-containing supplements due to the presence of other bioactive compounds such as tannins, theobromine, and catechins. It is considered that these effects are a result of the synergies between various bioactive compounds found in guarana [8,9,10]. Studies conducted on guarana showed that the plant improves the performance of tasks, working memory, and attention [1,11,12,13]. Most studies have found primary benefits in terms of response time and performance accuracy in the execution of tasks. Other studies have also found significant improvements in the secondary memory factor [1,14]. Some of the main reasons for the popularization of guarana are its cognitive effects, explained by the high content of methylxanthine (theophylline and theobromine) and caffeine, which is present in guarana seeds [8].

Guarana beans contain about 3.6–5.8% caffeine, compared to 1–2% in coffee beans [15]. Caffeine, structurally identified as 1,3,7-trimethylxanthine, is the most consumed psychoactive chemical in the world, being classified among the methylxanthine compounds. One of the main reasons for guarana’s widespread global use is its amount of caffeine, as it improves cognitive capacity and physical endurance to fatigue [14]. The caffeine content in guarana is significantly higher (about 2–4 times) than that in coffee, 30 times higher than in cocoa, and 10 times greater than that of yerba tea, all other popular stimulating beverages [3,4,11]. The pure form of guarana can contain up to 5.3% caffeine. In comparison, typical sources such as espresso coffee and dark chocolate have lower contents of around 0.21% and 0.08%, respectively [9]. The guarana caffeine is called guaranine, which the plant uses as a defense mechanism against herbivorous animals [1]. The caffeine content in guarana supplements is an important factor in their design. Several studies found no difference in memory and attention compared to a placebo. Later studies showed improvements in mood, emotions, and secondary memory. Additional studies have focused on the effects of guarana ingestion on cognitive performance, with some suggesting benefits and others finding no effect [9,11]. Guarana has evolved into a popular supplement due to its cognitive and physical enhancement properties. It can be consumed in a naturally infused or powdered form, or used in food like protein shakes and yoghurts. Caffeine, the stimulant found in this plant, is also used in energy drinks [11,16].

The chemical components found in the fruit of guarana (*Paullinia cupana*) include stimulants derived from methylxanthine (caffeine, theophylline, and theobromine) and tannins, compounds from interconnected monomeric units, the main being catechins and epicatechins. In addition, it is a good source of phosphorus, iron, magnesium, potassium, calcium, vitamin A, and vitamin B1. Guarana seeds have a high percentage of caffeine and lower proportions of other purines (theobromine and theophylline). They also contain a high concentration of polyphenols, especially proanthocyanidins. There are other substances with therapeutic properties present in guarana. Effects such as antioxidant and anti-inflammatory activity are due to increased concentrations of phenolic compounds [1,15,17]. Of the therapeutic properties due to high concentrations of phenols, inhibition of thrombocyte aggregation in vitro and in vivo is the most important. Guarana is considered a rich source of bioavailable catechins, producing measurable effects on oxidative stress, protecting the DNA of erythrocytes, and reducing the oxidation of the density of low-fat lipoprotein (LDL) [1,17]. Guarana seeds contain methylxanthine derivatives, which have been shown to have various physiological effects on the human body, including the nervous, respiratory, and cardiac systems. They stimulate skeletal muscle and promote diuresis. Methylxanthines can stimulate lipolysis and inhibit adipogenesis through multiple molecular mechanisms, contributing to obesity management by promoting fat breakdown and weight loss. Most of the health effects of guarana are related to its high antioxidant potential, which is attributed to the presence of tannins and catechins [3]. The nutritional composition of guarana seeds is estimated to comprise approximately 2–6% caffeine, 60% starch, 15% protein, 0.16% lipids, 14% phenolic constituents, 13% tannins, and 5.72% condensed tannins [1].

Guarana is a plant that exhibits psycho analeptic activity and has weight-loss properties. Studies have highlighted its protective effects against hypertension, obesity, and metabolic syndrome in healthy older volunteers, with a therapeutic role in atherosclerosis as well. Guarana modulates mitochondrial RNA and genes involved in adipogenicity processes, which increases energy metabolism and stimulates mitochondrial biogenesis. Its methylxanthine content may lead to changes in lipid metabolism, but these effects are linked to the plant’s tannin content. Guarana’s pharmacological effects are indeed primarily associated with its tannin content, which makes up 16% of the plant seeds’ composition. Additionally, guarana has been used for its diuretic, calming, and tonic effects [6]. Guarana consumption has been associated with enhanced cognitive function during exercise and at rest, potentially due to stimulants such as caffeine, flavonoids, saponins, or tannins. It is important to state that these findings are not conclusive, and further research is needed to fully understand the effects of guarana on cognitive function [18].

The stimulating effects of guarana persist longer than those of coffee, as caffeine in guarana binds to tannins. This is why it is added to a variety of energy drinks. Long-term consumption of the various components of these energy drinks can lead to significant changes in the cardiovascular system and even to seizures [6].

Guarana powder is readily available both in physical stores and online, and can be sold either individually or combined with other herbal medicines. It is also added to other types of food, such as athletes’ food, vitamin and mineral supplements, and functional foods. Guarana has a range of properties, including cytoprotective, hepatoprotective, neuroprotective, chemopreventive, anticancer, anxiolytic, colonic protector, antidepressant, antioxidant, and antimicrobial. Guarana-based supplements are marketed for their stimulating properties, including cognitive effects and weight loss [10]. In 2020, a Brazilian dietary supplement law recognized that guarana contains bioactive substances, thus validating its role as a functional food ingredient. Human studies found that the consumption of guarana powder was safe and did not cause toxicity in low doses (200 mg/day for humans) [19].

*Camellia sinensis* (green tea), commonly known as green tea, has a long history of medicinal use in China. The leaves of the plant can be processed in various ways to obtain black, green, or white tea. Green tea extract contains theanine, catechins, and flavanols, as well as caffeine. It has been found to have cardiovascular health benefits, but its effects on mood and cognition have been less studied [15,20].

*Camellia sinensis* grows in certain tropical and subtropical regions. There are four main subclasses of tea produced from the same plant, depending on how the leaves are processed: white, green, oolong, and black. Green tea is produced from the ripe leaves of this plant, with minimal processing, only by drying [21,22]. In Asia, green tea is the most consumed product, accounting for almost 20% of global tea production [21]. Studies have shown that green tea can improve blood circulation, lower cholesterol levels, prevent cardiovascular problems, and protect against the harmful effects of a high-fat diet. Additionally, green tea can promote relaxation and calmness, unlike soft drinks or liqueurs [7].

The primary medically relevant components in green tea are polyphenols, with the most important being flavonoids. Catechins are the most relevant flavonoids, representing 80–90% of all flavonoids and about 40% of the water-soluble solids in green tea. The amount of catechins in tea can be influenced by the harvesting and processing of the leaves, as well as the preparation of the drink [21].

Green tea is a functional food rich in beneficial components, such as proteins (15–20%), enzymes, carbohydrates (5–7%), less than 1% lipids, vitamins (B, C, and E), minerals (calcium, magnesium, chlorine, iron, zinc, selenium, sodium, potassium, aluminum), sterols, polyphenols (catechins 30–42%, caffeine 3–6%), theophylline, pigments, volatile compounds, and trace elements. Polyphenols, including catechins (flavan-3-ols) are the key therapeutic ingredients. There are four major catechins present in green tea: epigallocatechin gallate (EGCG), epigallocatechin (EEGC), epicatechin-3-galate (ECG) and epicatechin (EC) [23]. EGCG represent almost 59% of the total catechins, followed by EGC (approx. 19%), ECG (approx. 14%), and EC (approx. 6%) [21]. Compared to black tea and oolong tea, green tea contains a higher amount of polyphenols, such as EGCG, with neuroprotective action due to its high antioxidant activity [15,24]. Green tea as a dietary intervention may enhance cognitive function and mental clarity due to its high levels of EGCG [25,26]. Scientific studies have indicated beneficial effects of consuming green tea on overall health and reducing the risk of developing other diseases. Promising and positive results have been seen in the management of body weight control, protection against the action of ultraviolet radiation, physical functional performance, oral and bone health, and other physiological effects [27]. Studies on the beneficial effects of catechins from green tea have shown that it has anti-inflammatory and antioxidant properties. Additionally, it has been found to have anticancer, antimicrobial, anti-obesity, and antidiabetic properties. Green tea and its components, including catechins, may be a safer option for treating various diseases, such as cancer, diabetic warts, and cardiovascular and anogenital diseases [28]. Other studies demonstrated that the combination of green tea extract with L-theanine improved selective attention and memory performance and increased vigilance in people with mild cognitive impairment. A subsequent analysis determined that green tea consumption was associated with improved cognitive function in older people, but no conclusive link could be established [20,25,29]. Green tea consumption enhances cognitive function in elderly individuals, particularly in memory and executive function. It also reduces Alzheimer’s disease pathology and enhances antioxidative stress capacity. The protective effects are stronger with higher tea consumption [30]. Research results suggested that consuming at least 100 mL of green tea per day can lead to long-term benefits such as improved mental facilities, a more relaxed mood, and a lower risk of dementia. These effects seem to be dose-dependent, with the maximum effect being reached at 500 mL per day. Higher consumption of green tea has not been studied [31]. Supplementing with green tea in matcha form has been shown to significantly impact cognitive changes in female subjects, suggesting that daily supplementation could prevent cognitive decline in clinically normal elderly women [22]. As most studies suggest, green tea and its main ingredients, EGCG and L-theanine, can improve cognitive, neuropsychological, and other brain functions. Research results on daily and regular consumption of green tea, of at least 100 mL per day, suggest that the main long-term benefits are improved mental facilities, a more relaxed mood, and a lower risk of dementia [15,31,32,33].

This study investigated the potential of consuming green tea and guarana as an alternative to conventional stimulant products, such as coffee and energy or caffeine-based carbonated drinks commonly consumed by this category of the population, and their impact on students’ cognitive performance.

## 2. Materials and Methods

The effects of the consumption of guarana and green tea on the intellectual performance of students at Stefan cel Mare University in Suceava were tested. Prior to the commencement of the study, all participants were provided with comprehensive information regarding the nature, objectives, methodology, and anticipated outcomes of the research. The study was conducted in accordance with the Declaration of Helsinki, and approved by the Ethics Committee of Stefan cel Mare University of Suceava (no. 110/18.01.2023). This information was conveyed in an informed consent form signed by each participant. Furthermore, the participants were informed about the confidentiality measures applied to the data in accordance with the General Data Protection Regulation (GDPR) and were reassured that the results would be used solely for the purposes of the research project and would not affect their personal or professional lives in any way.

Thus, 33 students 19–25 years old participated in a randomized controlled study (Figure S1). One participant dropped out of the study due to health reasons. During the study, the participants consumed 2 g of guarana per day for 10 days, followed by a 72 h wash-out period. The study then continued for another 10 days, during which they consumed 1.5 g of green tea per day. The consumption option was chosen by each individual, but, based on the recommendations and information presented before enrollment in the study, consumption in the form of iced tea or shake was preferred. The aim of the study was to purchase safe products from recognized companies in the field of dietary supplement distribution. The study authors were responsible for the products supplied to the participants. Guarana, in the form of organic powder presented in 125 g packages, was portioned and distributed to the participants according to the study data. The green tea was distributed in single-use, individually wrapped packets. Before and after consumption, participants completed cognitive tests measuring information processing speed and response time, as well as questionnaires assessing cognitive, mental, emotional, and nutritional efficacy. These data were used to establish a baseline for later comparison of results. The first questionnaire consisted of a cognitive test tracking information processing/response speed, structured in three areas, distributed before and after consumption of green tea and guarana. This questionnaire was adapted in Romanian, after the DSST (Digit Symbol Substitution Test) [34]. The interpretation of the cognitive test is based on the calculation of scores, with one point awarded for each correct answer; wrong answers are not scored. As can be seen from the attached questionnaire, the numbers 1–9 are at the top of the page, with a graphic symbol associated with each of them. At the bottom of the test are random numbers with blank spaces below them. The task of the subjects was to identify the correct symbol for each number shown from left to right, without omitting any item. Participants were instructed to complete the task in 90 s after having previously viewed the numbers and corresponding symbols for 1 min. This test was performed before the study began and one and two weeks after consumption of the products, respectively. Multiple testing was avoided in order to prevent misinterpretation of the results due to the repetitive nature of the tests, and not based on the effectiveness of the neurostimulator effects of guarana and green tea consumption.

The second questionnaire was distributed after consumption of green tea and guarana, respectively. The first section requests information about participants’ current health and emotional status [35]. The second section includes items referring to consumer perception of sensory attributes of the products, purchase intention, and confidence in the products’ quality [35]. Section 3 contains questions on frequency of consumption, reason for and preferred place of consumption, and factors relating to organoleptic and functional product choice [35,36].

The last questionnaire was distributed after completion of consumption of both products, with the aim of identifying factors that contributed to the choice of consumption and which of the two products was easiest to prepare and consume [37]. As the final questionnaires represent parts of the validated questionnaires applied by researchers with experience in the field, the interpretation of the questionnaires took into account the way each section was calculated. The collected data were interpreted with Data Analysis Microsoft Excel 2010. The Likert scale was used to interpret the answers for all applied questionnaires, except for the cognitive test. The scale ranged from 1 to 5 (after green tea/guarana consumption) and from 1 to 4 (for the final version). The statistical analysis was interpreted using the Excel Stat trial version.

## 3. Results and Discussion

**Figure 1:**
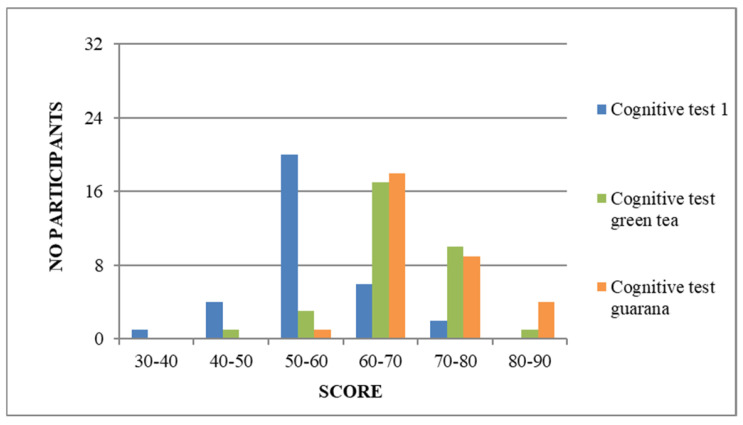
Scores obtained from the application of the cognitive test.

**Figure 2:**
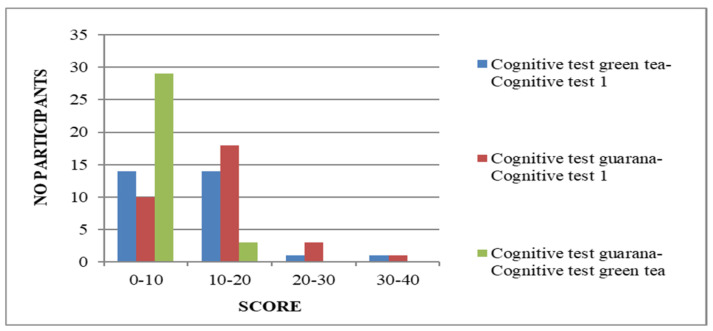
Differences in scores obtained in cognitive tests for guarana and green tea.

According to the data obtained from the cognitive tests’ results, the benefit of consumption can clearly be seen (Figure 1 and Figure 2).

If the initial cognitive test score was between 30 and 75, the highest score reached after consumption was 80. Additionally, the majority of participants in the study showed improved cognitive performance, with scores significantly higher than those of the initial test. For instance, during the initial phase, the minimum score required was 30. However, after consumption, the lowest score increased to over 40 points.

At the first completion of the questionnaire, the majority of students (n = 20) reached the 50-point threshold. In contrast, during the second meeting, the average score was 60 (n = 17). The lowest score on the first quiz was 39 points, while the highest was 75. In the second test, the minimum score increased to 49 points and the maximum to 80. Although green tea consumption resulted in increased speed and responsiveness for most students, guarana consumption showed a more significant difference in the 70–80 and 80–90 range compared to green tea. The test results showed that one participant scored in the 50–60 range, eighteen scored in the 60–70 range, nine scored in the 70–80 range, and four scored in the 80–90 range. The lowest score on the last test was 50 points, and the highest was 83. The scores showed significant differences, with an equal number of people (n = 14) having a difference of 0–10 and 10–20 points between the two tests. One person had a difference of 20–30 points, and another had a difference of 30–40 points. Two participants had a one-point drop in score between the initial and second tests, while one person maintained their score from the first test. On average, the score differences between the first and last test applied were in the range of 10–20 (n = 18), followed by 0–10 points (n = 10). Three students scored within 20–30 points of each other between the applied tests. One person scored significantly higher, with a difference of more than 30 points between the scores of the two tests. Additionally, two of the participants scored the same on both tests, while one individual achieved a score that was 2 points higher on the first test than the one completed after consuming guarana. To analyze the neurostimulator effects of the products, we considered the score differences (0–10 points) obtained from tests after consuming green tea and guarana. Out of 29 individuals, only three participants scored 10 and 20 units higher, respectively.

In their study, O. Kassis et al. examined the impact of consuming products with high caffeine content, including coffee, on energy levels. Using the same cognitive test as the present study, they found that the energizer was more effective in activating reaction-response speed than coffee consumption. The results of their study confirm that guarana and green tea have a greater positive impact on intellectual performance compared to coffee [34].

A study conducted by Kennedy on guarana, aimed at evaluation its efficacy on cognition employed several devices (CDR) and tests (visual, verbal, numbers), whereas the current study used a digit substitution test. Moreover, Kennedy’s supplemented dose was 75 mg guarana, compared to the current study, which used a dose of 2 g/day. The study had more noticeable results due to the differences in supplementation, dose, tests applied, and methods of analysis compared to this one, where a single cognitive method test was used. The study also compared the effect on participants of guarana with that of Panax ginseng, and with their combination, while the current study focused mainly on comparing the cognitive enhancing effects of guarana and green tea [13].

**Figure 3:**
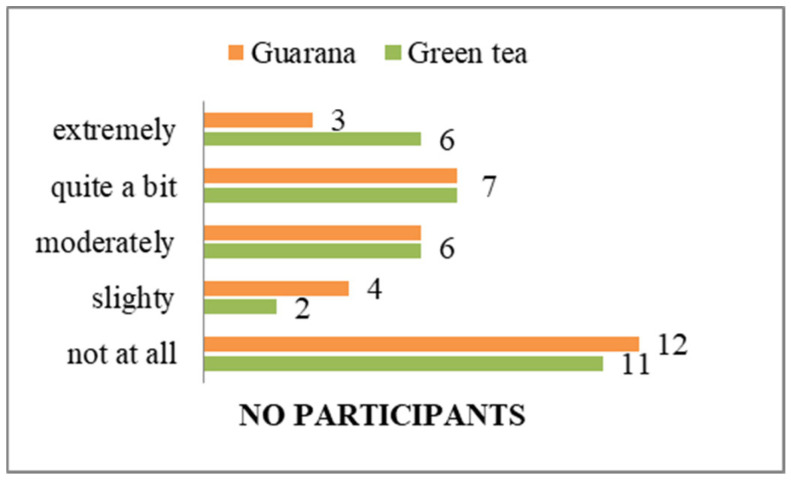
Impact of physical or mental health on normal activities.

The objective of including these questions in the initial section of the questionnaires was to ascertain the psycho-emotional state of each subject, in order to prevent any potential interference with the outcomes of the study. According to the responses, the majority of students do not perceive themselves to have any physical or mental health issues that could potentially impact their life (Figure S3) and believe that they would not experience physical or mental health issues that would impair their daily activities (Figure 3).

**Figure 4:**
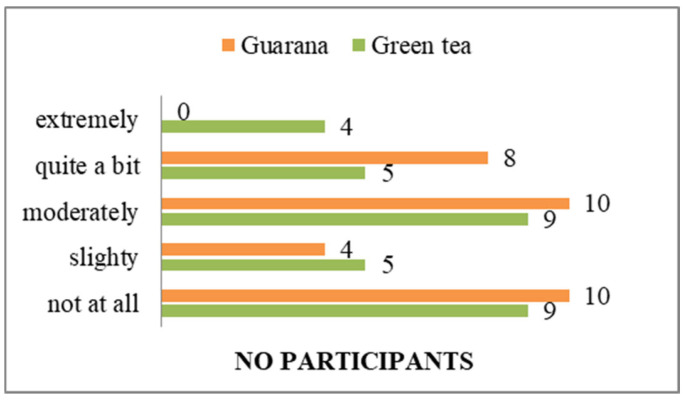
Level of concern about having a serious illness.

In their responses, they do not express concern or worries about the existence of pathologies (Figure 4).

**Figure 5:**
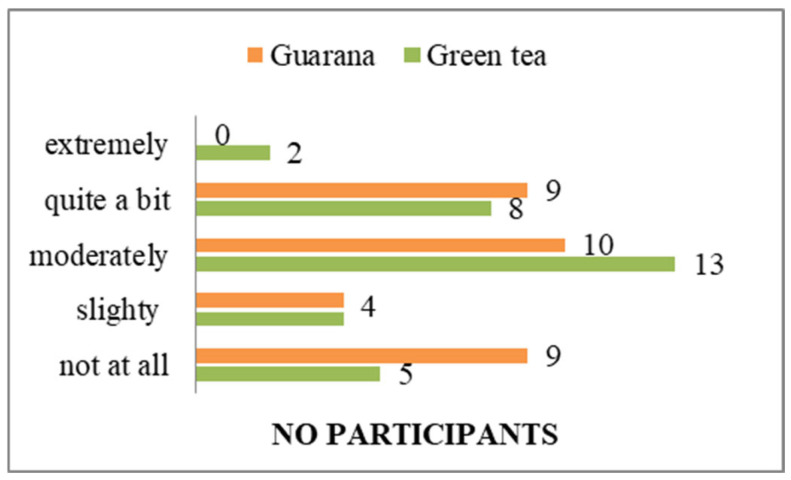
General concern about personal health.

**Figure 6:**
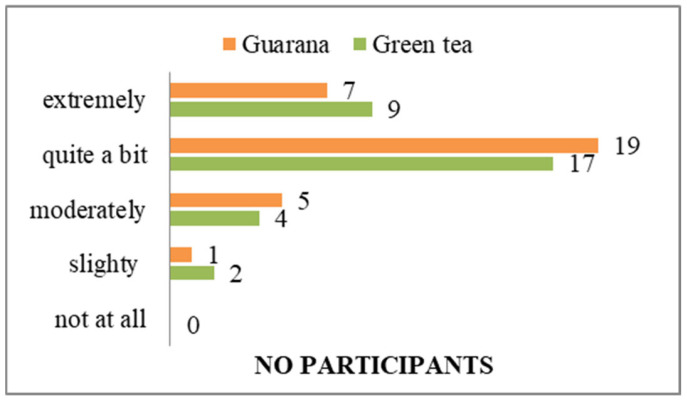
Understanding of illness symptom management.

**Figure 7:**
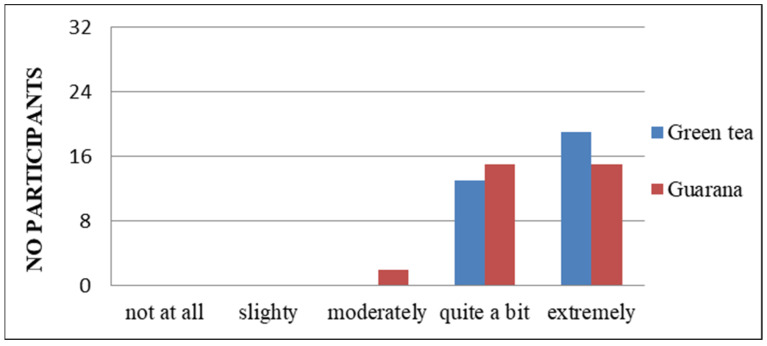
Perception of buying green tea/guarana as a right choice.

Additionally, the majority of students do not perceive their general state of health as a significant concern (Figure 5), and a notable proportion of them indicate that they possess knowledge about how to prevent the symptoms of potential diseases (Figure 4, Figure 6 and Figure S3). A total of 11 respondents (33%) demonstrated familiarity with information pertaining to the consumption and benefits of green tea. The majority of respondents expressed strong agreement with the use of green tea, whereas only seven indicated a similar level of endorsement for guarana. With regard to the information on guarana, five of the participants exhibited a neutral attitude, while the majority displayed a positive one (Figure S6). As illustrated in the figures, participants demonstrated a greater familiarity with green tea compared to guarana. The students understood the significance, the system, and the standards regarding the consumption of green tea and guarana (Figures S6 and S8). Furthermore, they indicated a strong preference for green tea and guarana, perceiving them as beneficial foods (Figure 7 and Figure S8) with high nutritional qualities (Figure S41).

The market for food supplements is experiencing continuous growth, particularly due to the increasing consumer preference for such products. It is therefore crucial that these products are certified and verified in terms of compliance and safety for consumption. The data obtained indicate that these issues are of significant interest to the participants included in the study. The majority of them expressed agreement or strong agreement (Figures S12–S14). Thus, the respondents expressed confidence in the veracity of the information presented on product packaging, as specified by the manufacturer or retailer. This is an important aspect in establishing consumer trust (Figure S16). They demonstrated a willingness to seek further information on these products (Figure S42). The results obtained from the questionnaire, completed by students after consuming green tea or guarana, indicate that the majority were satisfied with the effects observed and would be willing to purchase such products (Figure S15). Furthermore, the respondents expressed a willingness to recommend these products (Figure S16) and did not identify price as a primary determinant in their consumption of green tea or guarana (Figure S43).

**Figure 8:**
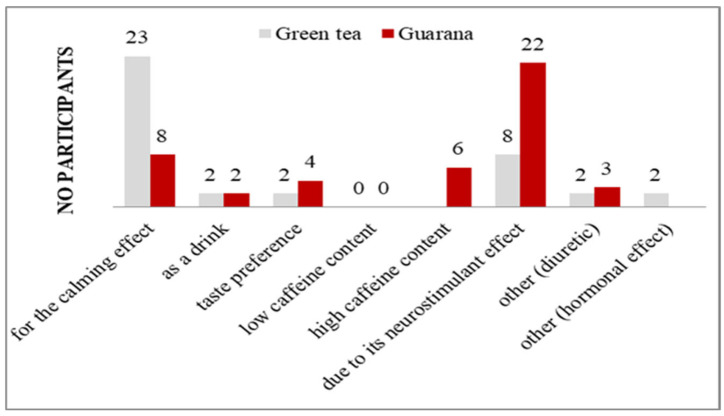
Reasons for consuming green tea/guarana.

A total of 28 individuals indicated that they consumed green tea on a daily basis, whereas 30 subjects reported consuming guarana daily (Figure S18). Subjects indicated a preference for the consumption of both products in the morning. However, in the case of green tea, there were students who preferred to consume it in the evening. It is noteworthy that a number of participants indicated a preference for an alternation of consumption intervals, contingent on the availability of time (Figure S19). With regard to the setting in which consumption occurs, 28 subjects, irrespective of the product consumed, indicated a preference for the home environment, although some exceptions were noted, namely the workplace, cafés, and the university (Figure S20). The individuals who consumed green tea cited a calming effect as the primary reason for consumption, while guarana consumers experienced a neurostimulatory effect (Figure 8).

The organoleptic and functional factors that informed the selection of green tea consumption were primarily taste and nutritional composition, as well as the benefits obtained. In the case of guarana, nutritional composition and benefits were of particular importance (Figures S21 and S22). The social factors and habits that led to the consumption of these products were mainly observed before eating a meal, for the purpose of enhancing concentration during task management, and after eating a meal. In the case of green tea, two participants indicated that they consumed it before bedtime, while two others cited it as an alternative for hydration following exercise (Figure S23).

**Figure 9:**
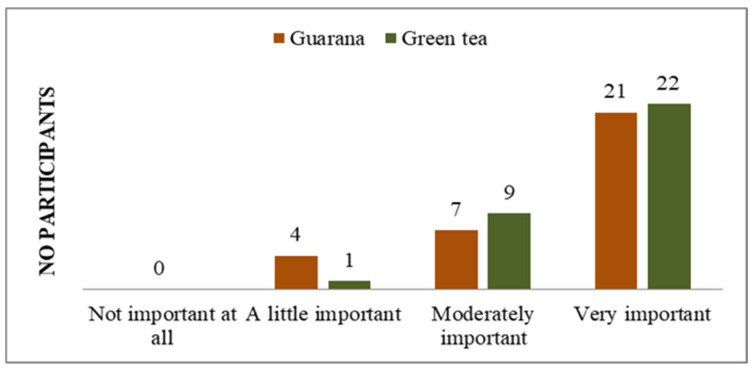
Anti-stress effects of green tea/guarana.

**Figure 10:**
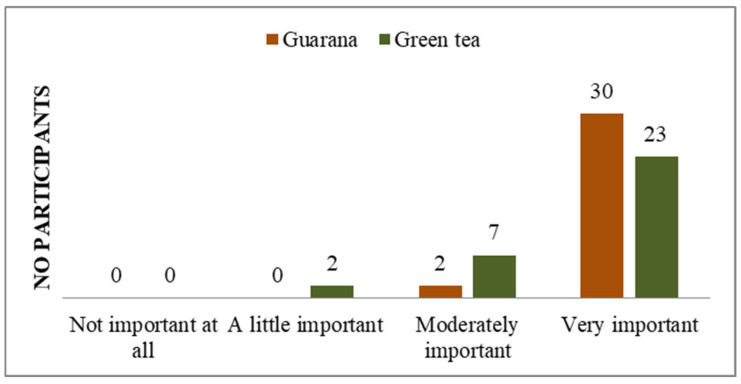
Effect of green tea/guarana on wakefulness.

**Figure 11:**
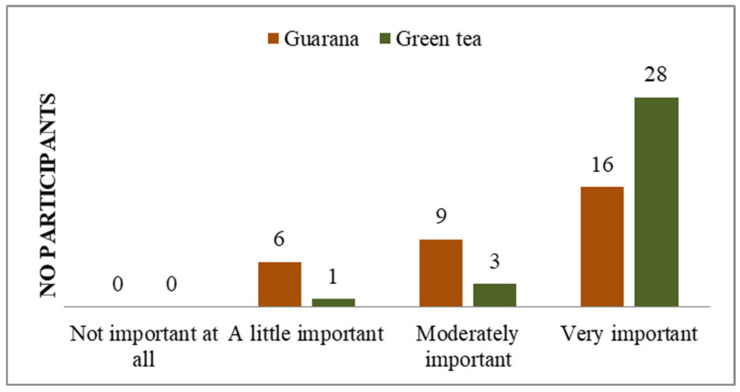
Relaxation benefits of green tea/guarana.

**Figure 12:**
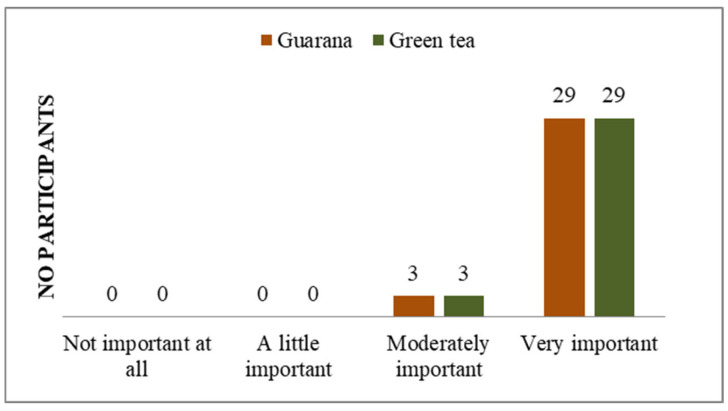
Health benefits of green tea/guarana consumption.

**Figure 13:**
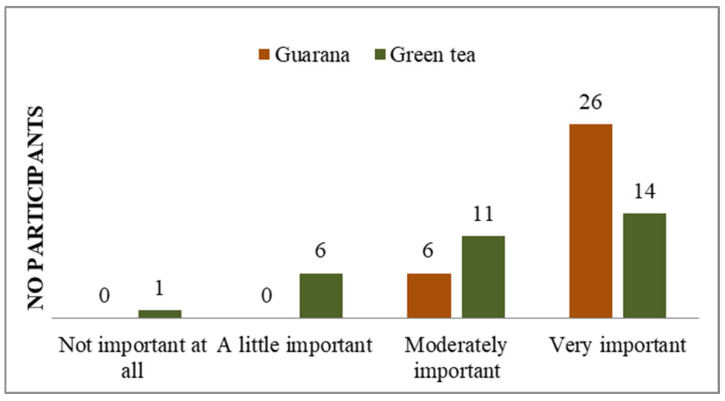
Neurostimulator effects of green tea/guarana.

In terms of organoleptic characteristics, the majority of students consider green tea to be a simpler product to prepare than guarana (Figure S24). Similarly, the same number of people consider both to be low in calories (Figure S25). However, green tea is perceived to taste better than guarana (Figure S26). Students agree with the information about green tea/guarana consumption, while an equal number (n = 5) have no opinion, and only one person in each category disagrees with the information. The results indicated that respondents have extensive information on green tea consumption, in contrast to guarana, for which some possess familiarity but less detailed knowledge than they do of green tea. Two individuals express disagreement regarding the ease of purchase, six have no opinion, and the majority (n = 24) agree (Figure S27). In terms of perceived wellbeing effects, all subjects indicated that they believe guarana to have a superior impact compared to green tea (two subjects answered neutrally) (Figure S28). The olfactory perception of green tea was found to be more agreeable than that of guarana, with one individual reporting an unpleasant olfactory sensation in response to the latter (Figure S29). Participants preferred the viscosity of guarana over that of green tea (Figure S30). From the perspective of product affordability, the majority of respondents indicated that green tea was a more accessible option than guarana (Figure S31). This situation could be improved if the consumption of guarana increases, thereby making it a product of interest for distributors. In terms of nutritional composition, 29 individuals indicated that both guarana and green tea contain natural ingredients (Figure S32). Additionally, 29 individuals perceived guarana to have a lower level of additives compared to green tea (Figure S33). The respondents also perceived guarana to have a lower fat content (Figure S34), and both products were considered to be high in fiber (Figure S35), protein (Figure S36), and vitamins and minerals (Figure S37). Although some of the students participating in the study indicated that they had no opinion on the matter (n = 9), the majority expressed agreement (n = 23) that both products contain a significant amount of polyphenols (Figure S38). In terms of the effects felt following consumption, the anti-stress effect was observed by respondents in the case of green tea (Figure 9). The energizing effect was particularly felt after guarana consumption, with 30 participants confirming its effectiveness (Figure 10). The calming effect was particularly felt after the administration of green tea, with 28 subjects agreeing (Figure 11). Based on the effects identified, respondents considered that both products positively influenced health (Figure 12). Based on the responses received, the majority of people agreed that green tea improves the health of hair, teeth, and nails (Figure S39). All students agreed that the products consumed helped to maintain quality of life, regardless of whether they consumed green tea or guarana (Figure S40). The neurostimulant effect was more pronounced with guarana than with green tea, with 26 individuals indicating that they agreed that guarana had effects on intellectual performance, compared to 14 responses for green tea (Figure 13).

In a study by Z. Szakály et al., the factors that contributed to the frequency of green tea consumption were health, nutritional composition, price, conviviality, and familiarity. In the present study, the primary factors influencing the acceptability of the product were those related to its nutritional value and the benefits it offered. Although the majority of respondents expressed a positive attitude towards the product, a number of exceptions were observed in relation to specific attributes, including energy content, composition, accessibility, taste, texture, and odor. In these instances, respondents demonstrated a neutral or negative response [38].

According to the data obtained in terms of the cognitive tests, it can be observed that the *p*-value indicates statistical significance (*p* < 0.0001) in all the tests (before the study, after green tea consumption, and after guarana consumption). These results indicate that there is a significant association between guarana consumption and improved cognitive performance (*p* < 0.0001), whereas green tea consumption does not appear to provide a similar benefit (*p* = 0.248). This suggests that guarana may be a potential source of cognitive performance enhancement and may merit further investigation in this regard.

A limitation of the study is that these products should be consumed over an extended period and tested on a larger and more diverse sample for more conclusive and tangible results.

The main strength of the study lies in the results obtained. Although the study group included a small number of participants, they agreed that both guarana and green tea consumption were beneficial to their health and helped them in their orientation towards purchasing healthy dietary supplements over beverages such as energy drinks or other stimulant products. However, it is important to state that the uneven sample distribution between genders may be a limitation of the current study. The higher percentage of females compared to males may induce a potential bias, and the findings may not be fully generalizable to a more balanced population. Future research with equal gender distribution in order to enhance the validity of the results should be taken into consideration. Following consumption, brain-level devices can be used to analyze the benefits of these products on responsiveness, speed, and accuracy during memory exercises. The tests should also be interpreted by specialists with expertise.

## 4. Conclusions

The present study investigated the effects of guarana and green tea consumption as alternatives to stimulant drinks frequently consumed by students, such as coffee and energy drinks or carbonated drinks with high caffeine content. The results of the cognitive test indicated an increase in the ability to respond to requests after the consumption of green tea and guarana. Throughout the study, the consumption of guarana and green tea presented neurostimulating effects, based on the results obtained from the cognitive tests applied. However, it was found that guarana produces better results in terms of intellectual performance, compared to green tea. The physical and mental state of the students included in the study was identified before enrollment, through tests and questionnaires applied. Any doubt regarding these questions meant the elimination of the participant from the study. After completion of the questionnaires by the study participants, it was found that green tea has stronger calming effects and provides a feeling of well-being among students. Guarana, in addition to its neurostimulating effect, also demonstrated other beneficial effects among the subjects, such as increased energy levels and better concentration. The results obtained are of interest and highlight the possibility of these substances being consumed as alternatives to drinks frequently chosen by students, such as energy or carbonated beverages with a high content of carbohydrates, caffeine, and other harmful chemicals. Often preferred by students during periods of physical and intellectual effort, but also during meetings with friends, these drinks, recognized worldwide as harmful to health, can be replaced with those studied in this work. Furthermore, the positive results obtained after testing can offer good examples for students, as was the case among those included in the study group.

## Data Availability

The original contributions presented in this study are included in the article. Further inquiries can be directed to the corresponding author.

## Supplementary Materials

The following supporting information can be downloaded at: https://www.mdpi.com/article/10.3390/nu17061000/s1.

## Abbreviations

The following abbreviations are used in this manuscript:

DNA	Desoxyribonucleic Acid
EC	Epicatechin
ECG	Epicatechin-3 -gallate
EGC	Epigallocatechin
EGCG	Epigallocatechin-3-gallate
LDL	Low-Density Lipoprotein
RNA	Ribonucleic Acid
RNS	Reactive Nitrogen Species
