# Supplementary material for: Effects of Guarana and Green Tea Consumption on Students’ Intellectual Performances
Source: Nutrients. 2025 Mar 12;17(6):1000. doi: 10.3390/nu17061000 (PMC11945102; doi:10.3390/nu17061000)
Supplement: Supplementary file 1 [file nutrients-17-01000-s001.zip › nutrients-3454844-supplementary.pdf]

Supplementary file

"Effects of guarana and green tea consumption on students' intellectual performances"

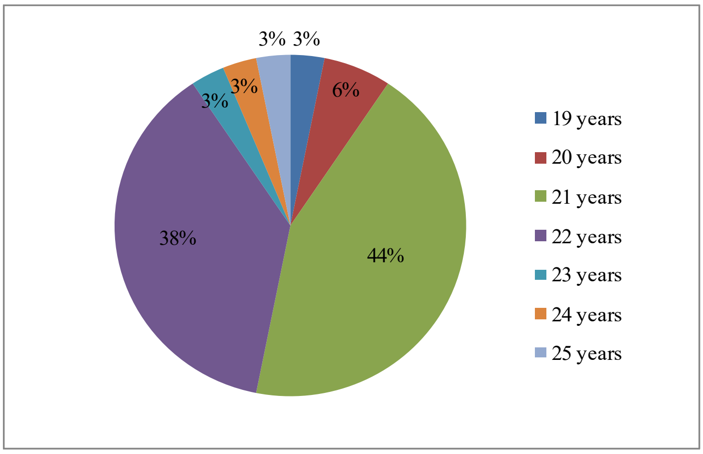

Figure S1. Participants' ages

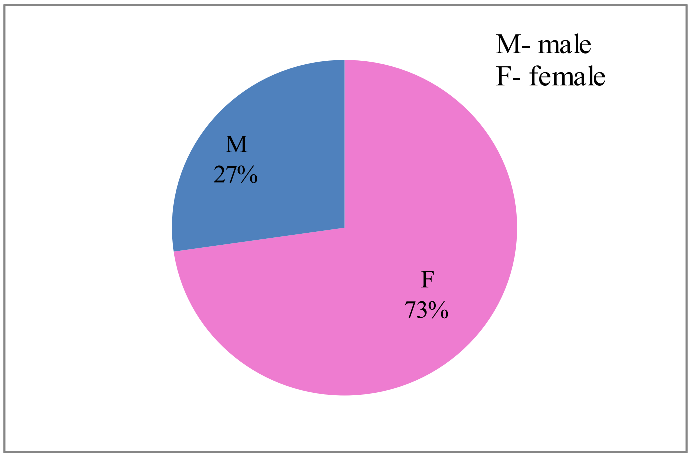

Figure S2. Gender of students involved into research

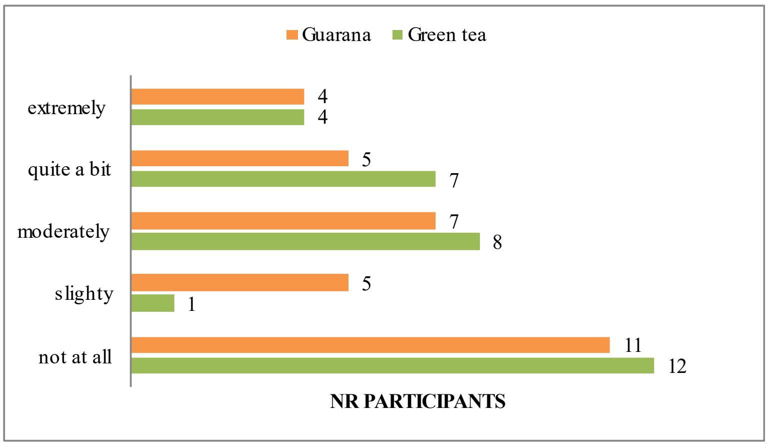

Figure S3. Impact of Physical or Mental Health on Enjoyment of Life

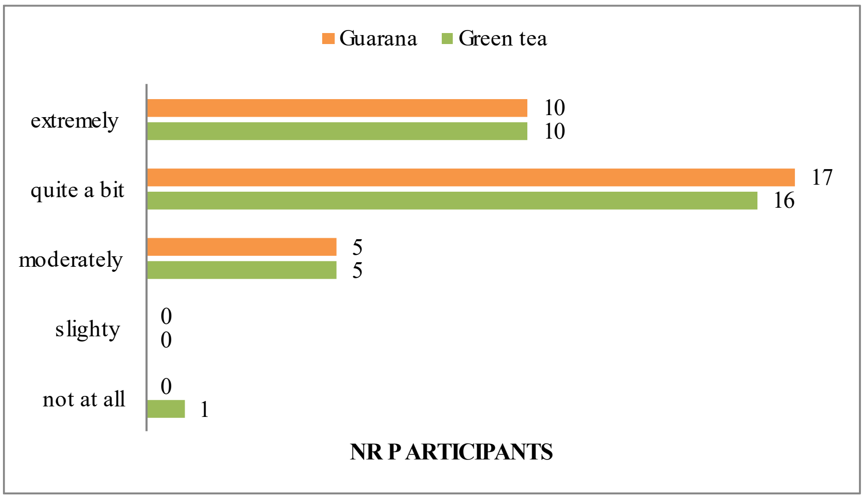

Figure S4. Understanding of Current Illness or Health Problems

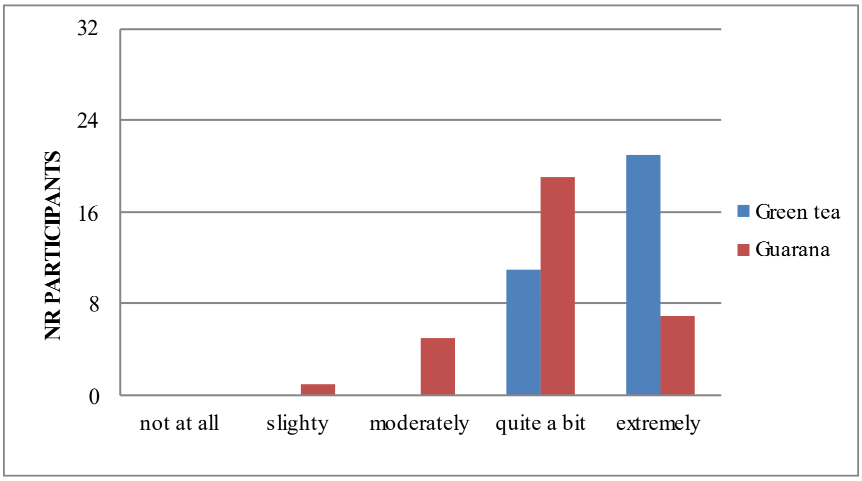

Figure S5. Familiarity with the Term Green Tea/Guarana

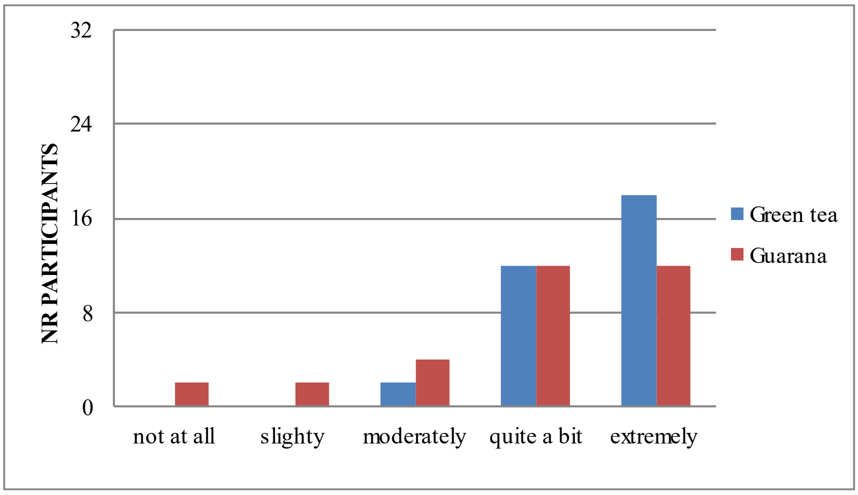

Figure S6. Understanding the Meaning of Green Tea/Guarana

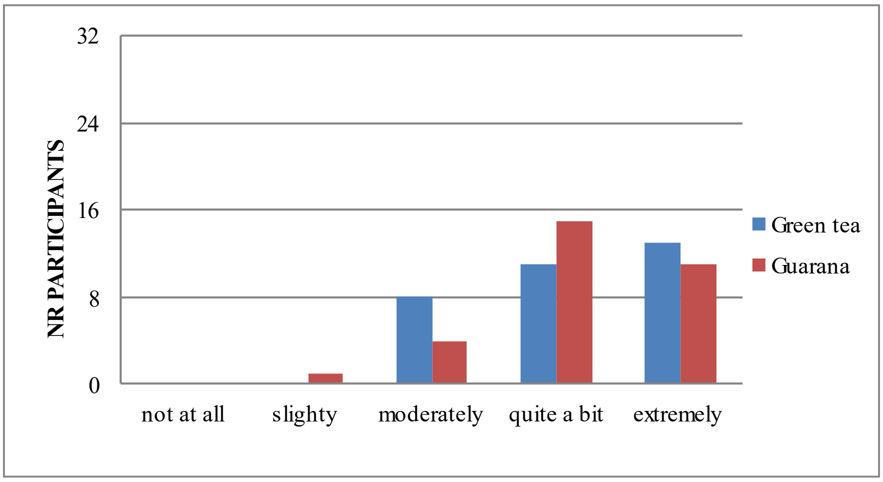

Figure S7. Understanding of Systems, Standards, and Information Related to Green Tea/Guarana

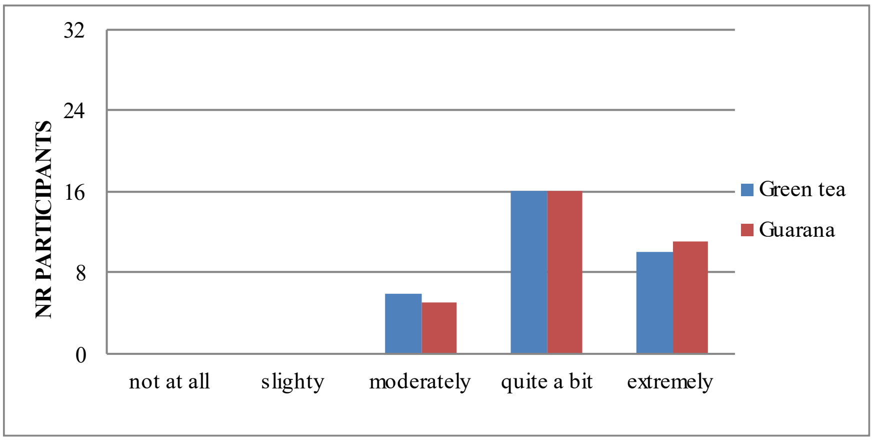

Figure S8. The Role of Green Tea/Guarana in Avoiding Nutritionally Unhealthy Products

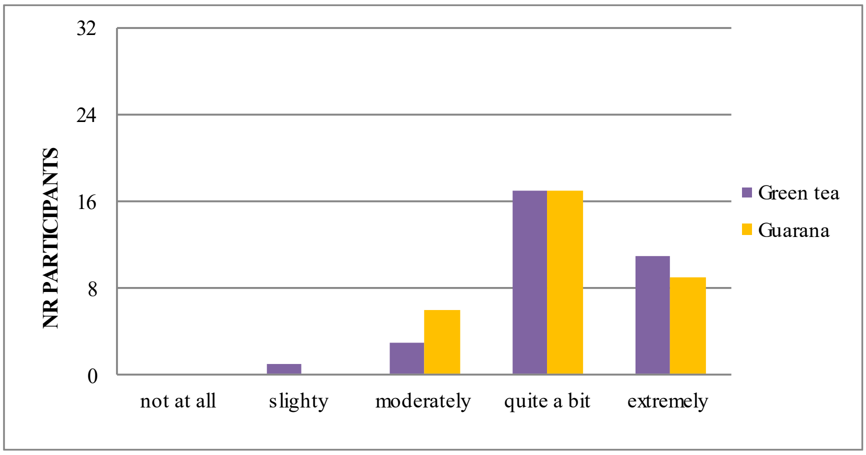

Figure S9. Perception of the Quality and Safety of Green Tea/Guarana

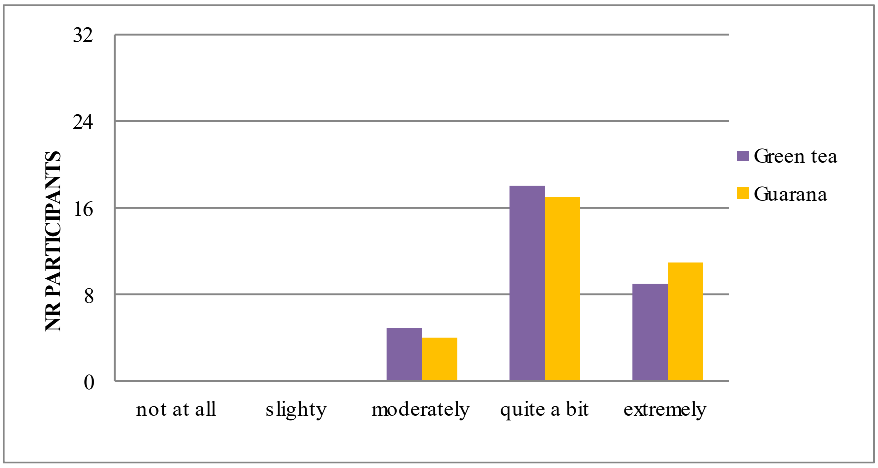

Figure S10. Perception of Green Tea/Guarana's Environmental Impact and Alignment with Sustainable Development

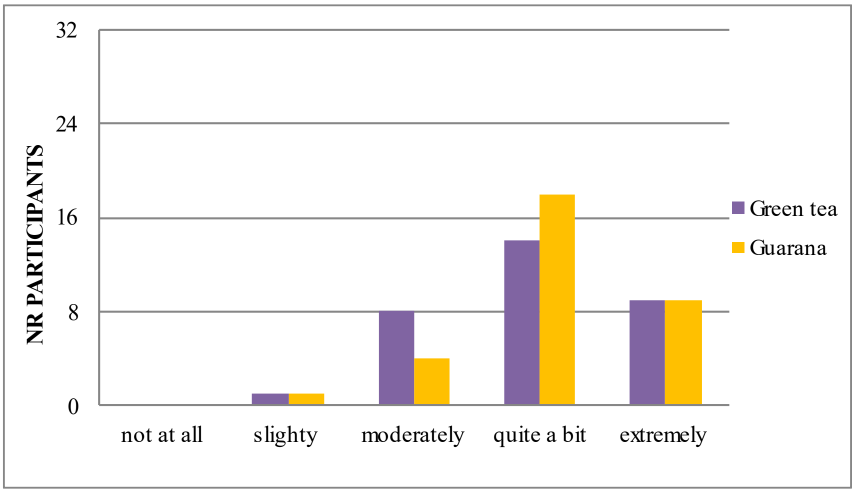

Figure S11. How Green Tea/Guarana Meets the Needs

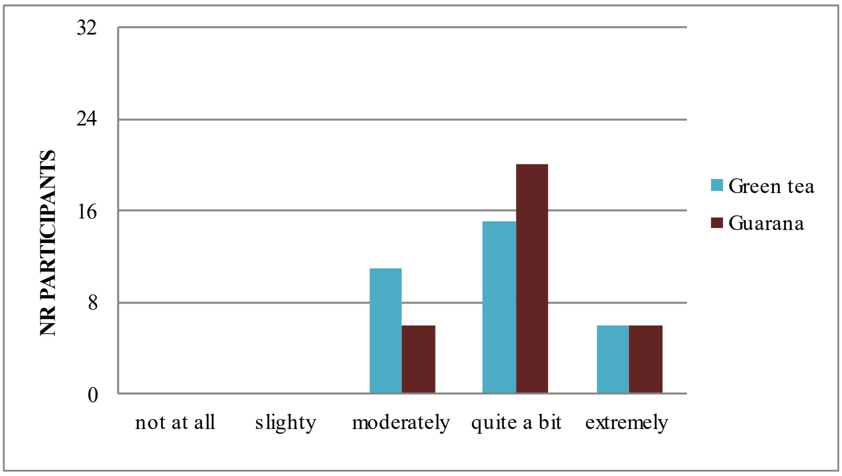

Figure S12. Perception of the Credibility of Green Tea/Guarana Certification Organizations

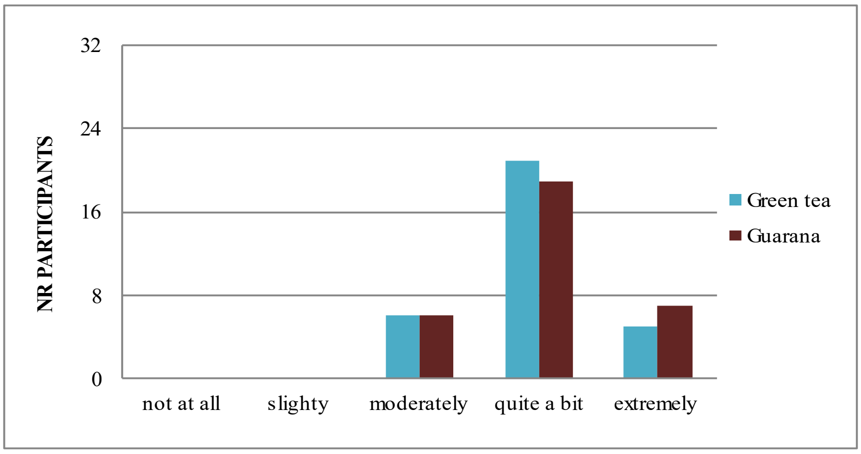

Figure S13. Belief in the Fairness and Credibility of the Green Tea/Guarana Certification Process

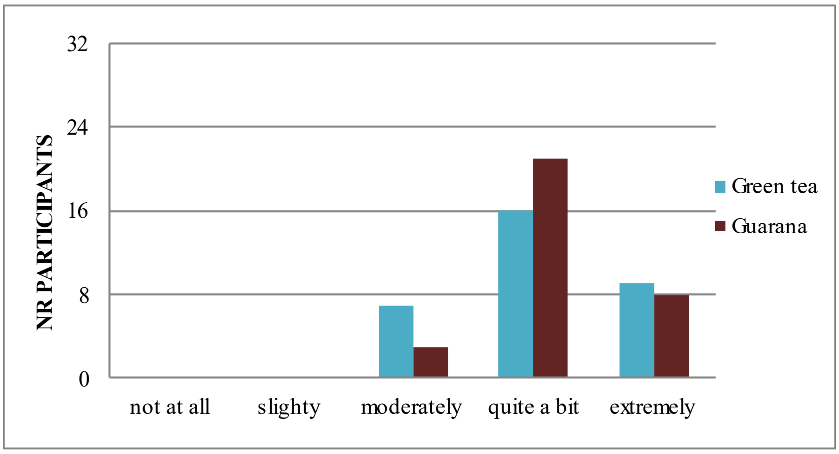

Figure S14. Belief in the Truthfulness of Information Provided by Green Tea/Guarana

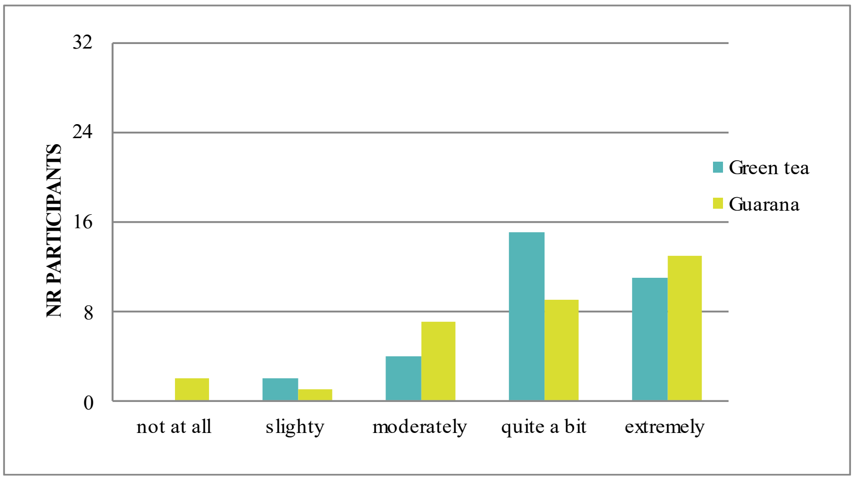

Figure S15. Consideration of Purchasing Green Tea/Guarana

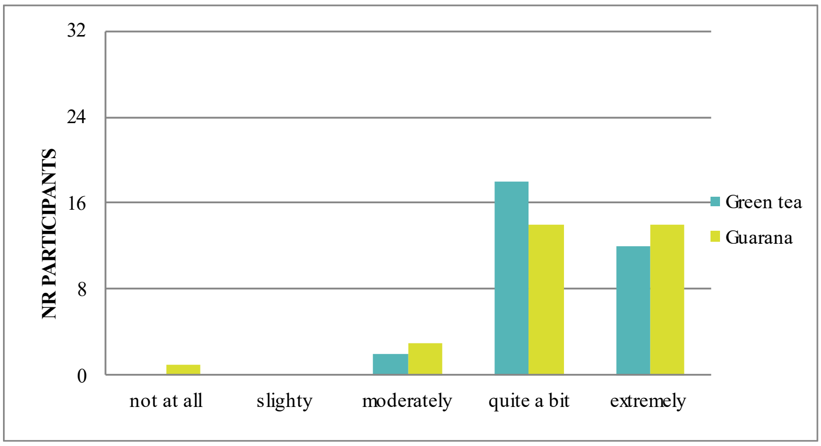

Figure S16. Willingness to Recommend Green Tea/Guarana to Others

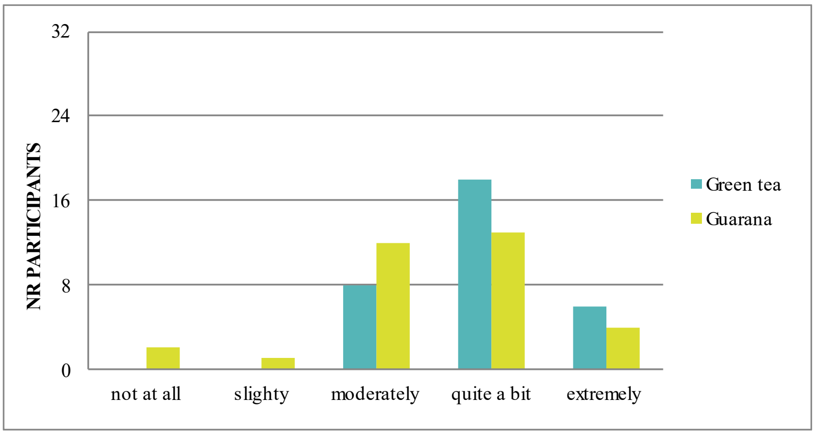

Figure S17. Anticipated Increase in Frequency of Purchasing Green Tea/Guarana

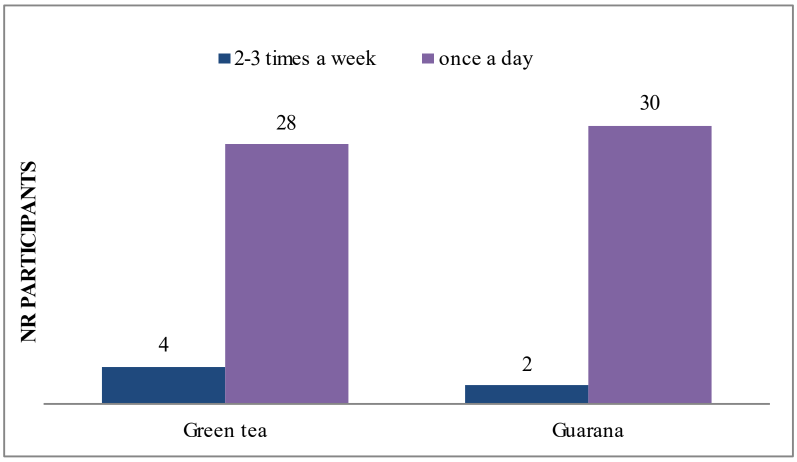

Figure S18. Frequency of Drinking Green Tea/Guarana

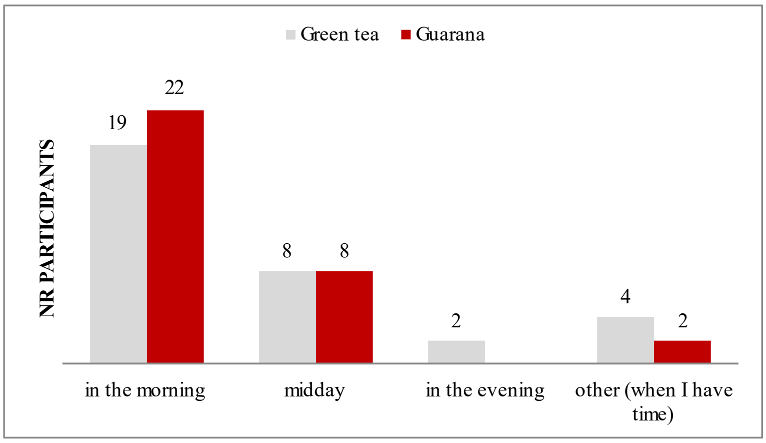

Figure S19. Typical Times for Drinking Green Tea/Guarana

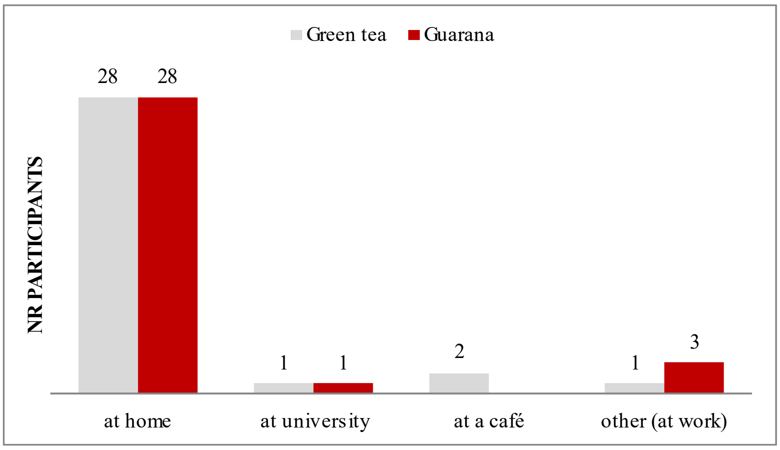

Figure S20. Typical Locations for Drinking Green Tea/Guarana

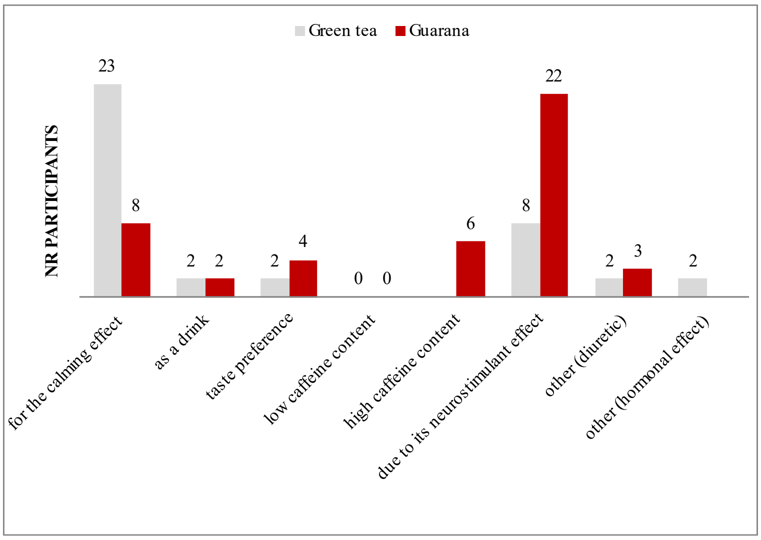

Figure S21. Reasons for Drinking Green Tea/Guarana

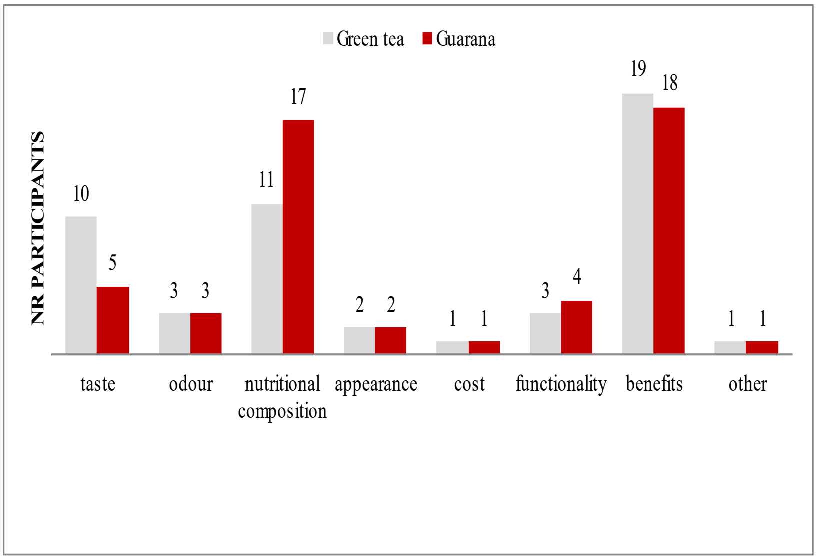

Figure S22. Factors Contributing to the Choice of Green Tea/Guarana Consumption

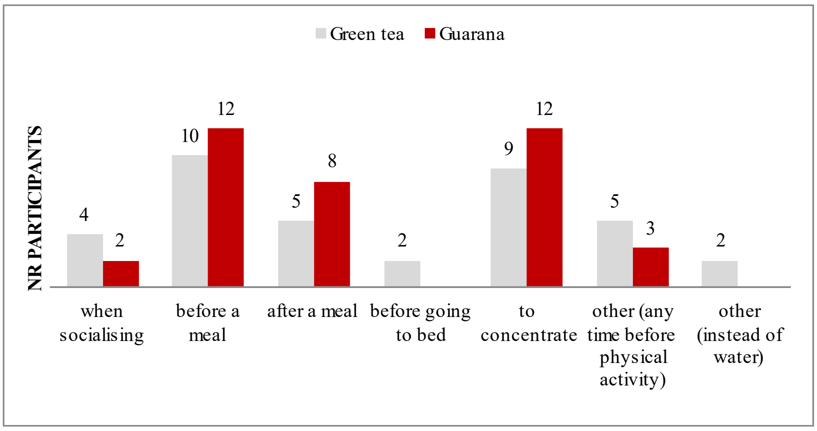

Figure S23. Preferred Situations for Drinking Green Tea/Guarana

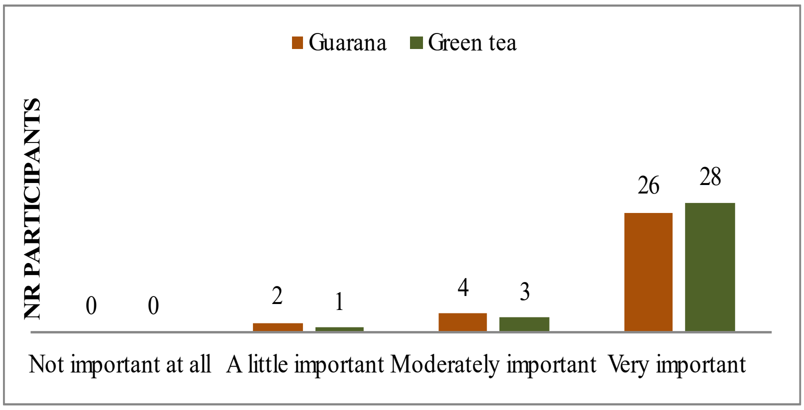

Figure S24. Perceived Ease of Preparing Green Tea/Guarana

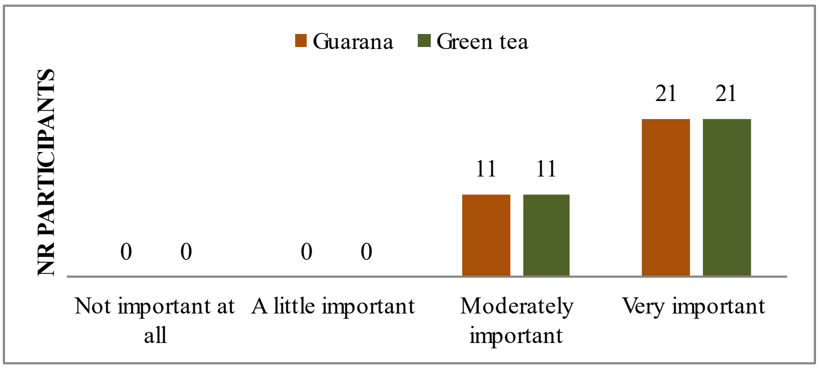

Figure S25. Perception of Green Tea/Guarana as Low- Calory Product

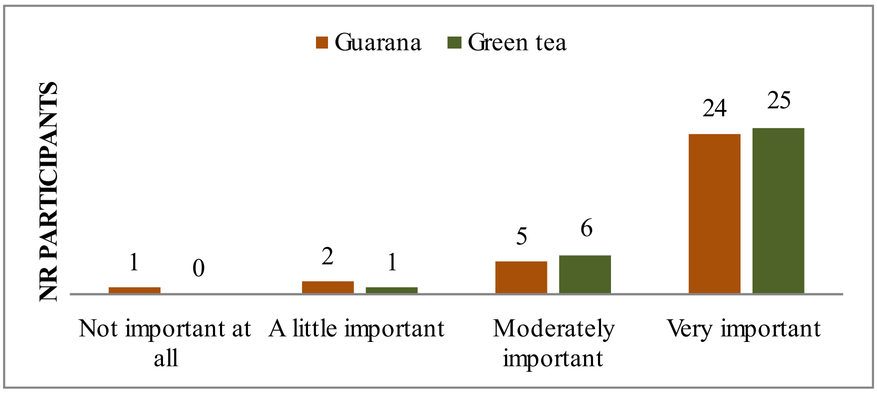

Figure S26. Perception of the Good Taste of Green Tea/Guarana

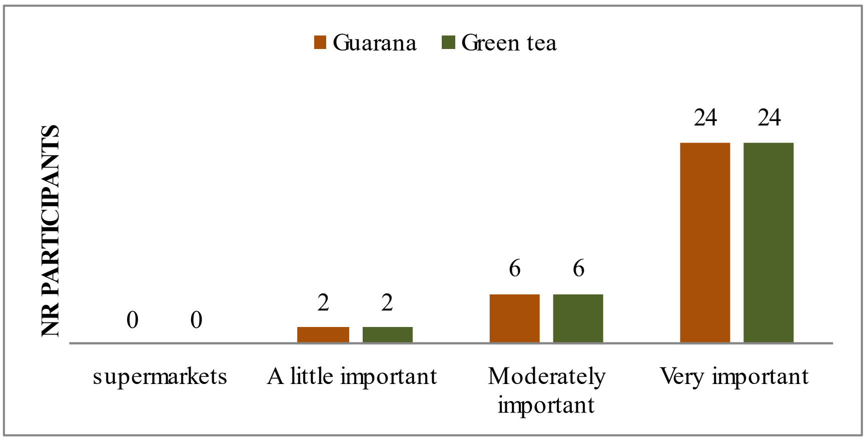

Figure S27. Availability of Green Tea/Guarana in Shops

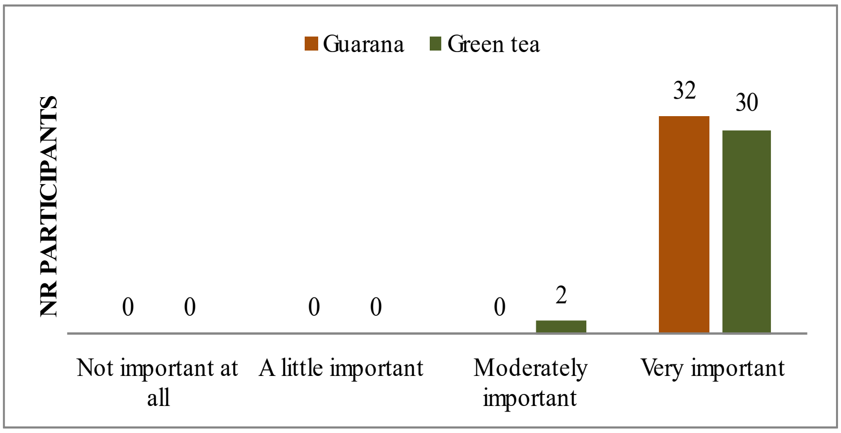

Figure S28. Perception of Green Tea/Guarana as a Mood Booster

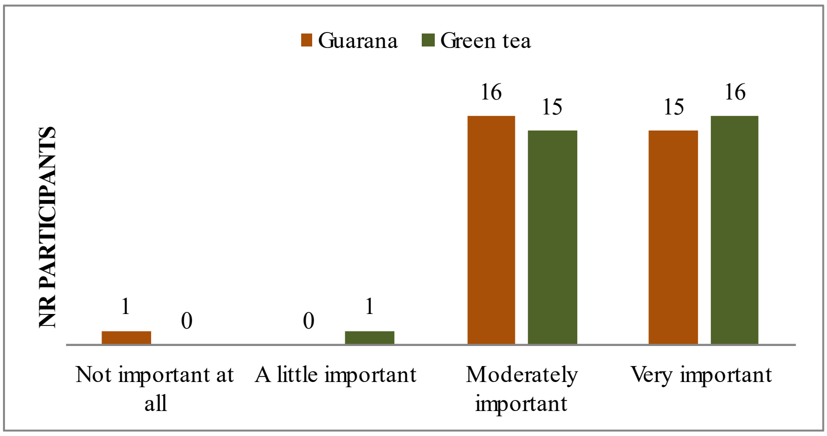

Figure S29. Perception of the Good Smell of Green Tea/Guarana

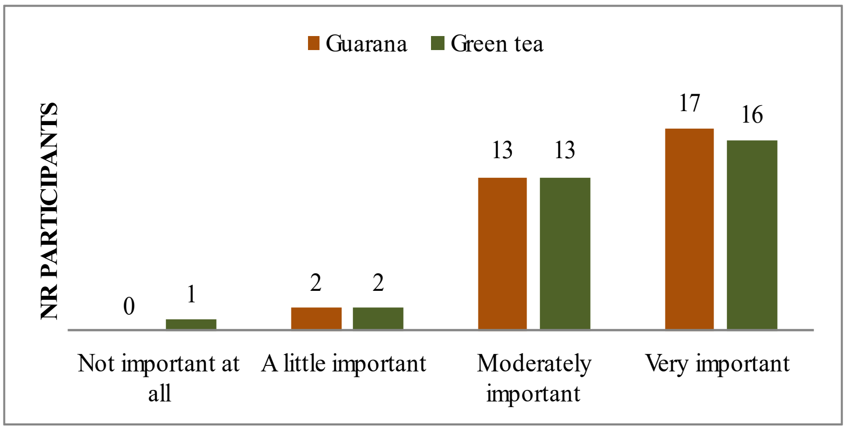

Figure S30. Perception of the Pleasant Texture of Green Tea/Guarana

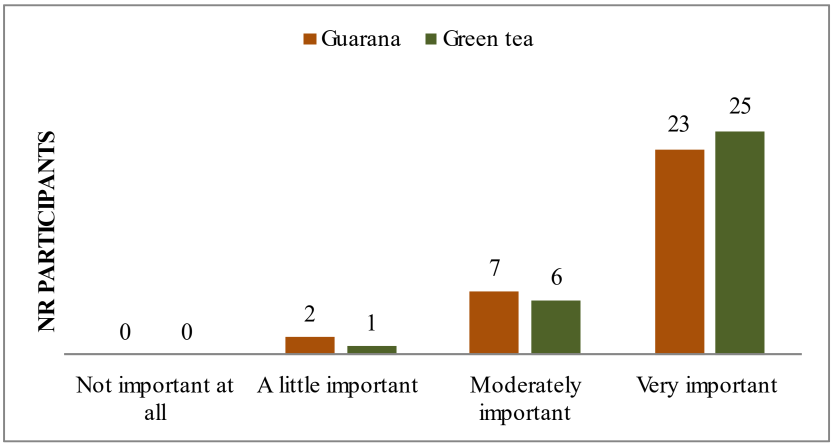

Figure S31. Perception of Green Tea/Guarana as Good Value for Money

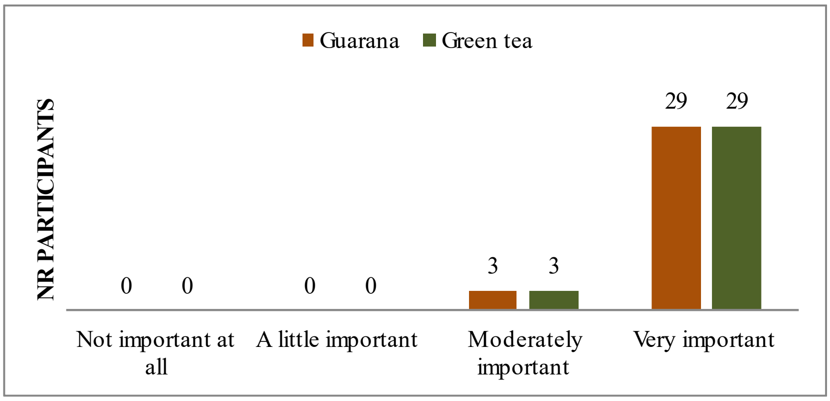

Figure S32. Perception of Green Tea/Guarana Containing Natural Ingredients

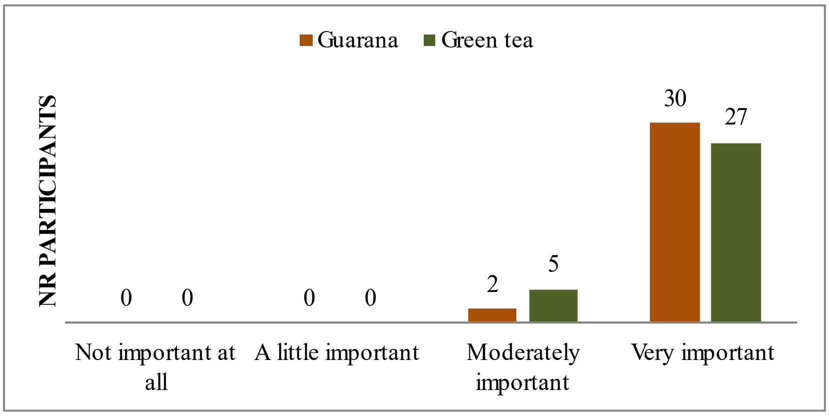

Figure S33. Perception of Green Tea/Guarana as Containing No Additives

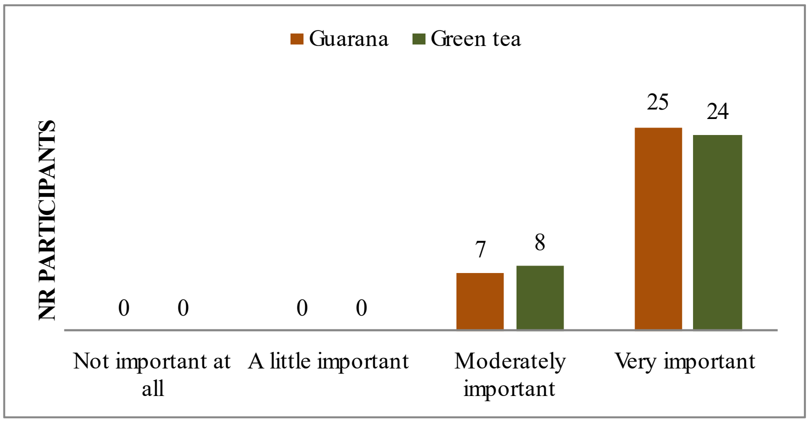

Figure S34. Perception of Green Tea/Guarana as Low in Fat

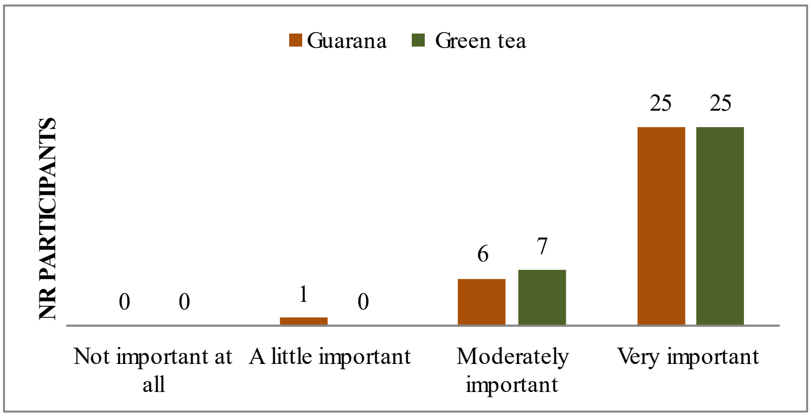

Figure S35. Perception of Green Tea/Guarana as Rich in Fiber Product

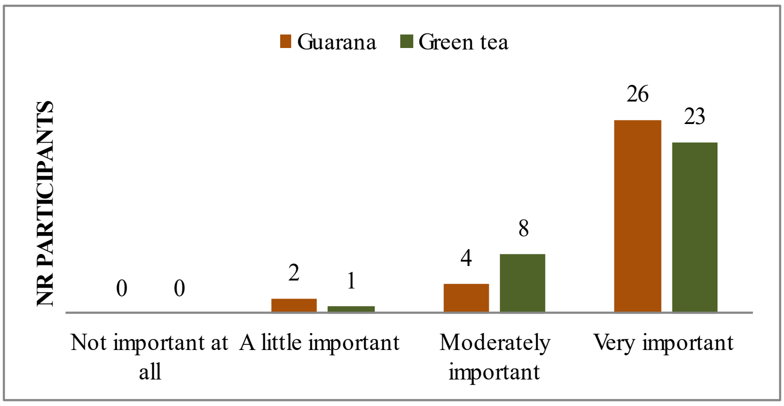

Figure S36. Perception of Green Tea/Guarana as Rich in Protein Product

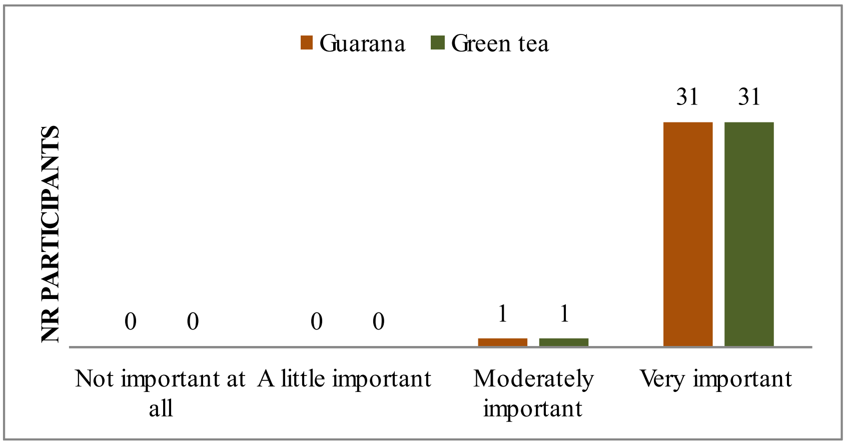

Figure S37. Perception of Green Tea/Guarana as Containing Lots of Vitamins and Minerals

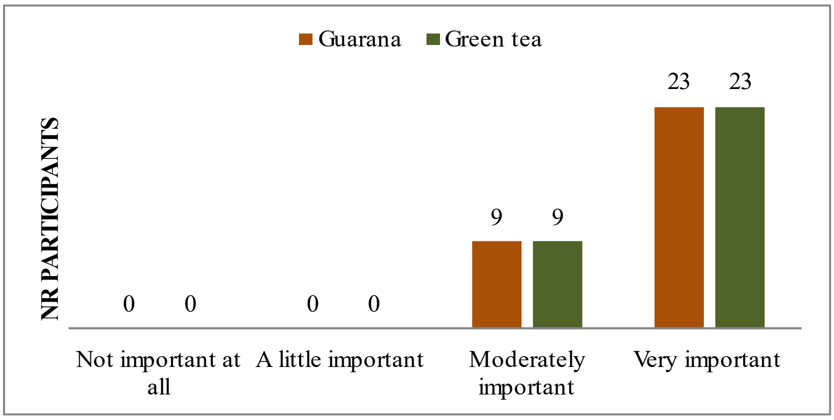

Figure S38. Perception of Green Tea/Guarana as Containing Polyphenols

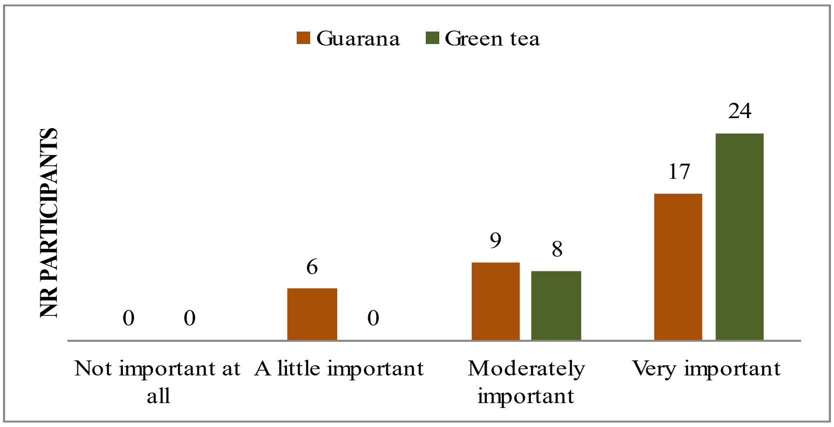

Figure S39. Perception of Green Tea/Guarana as Beneficial for Skin, Teeth, Hair, and Nails

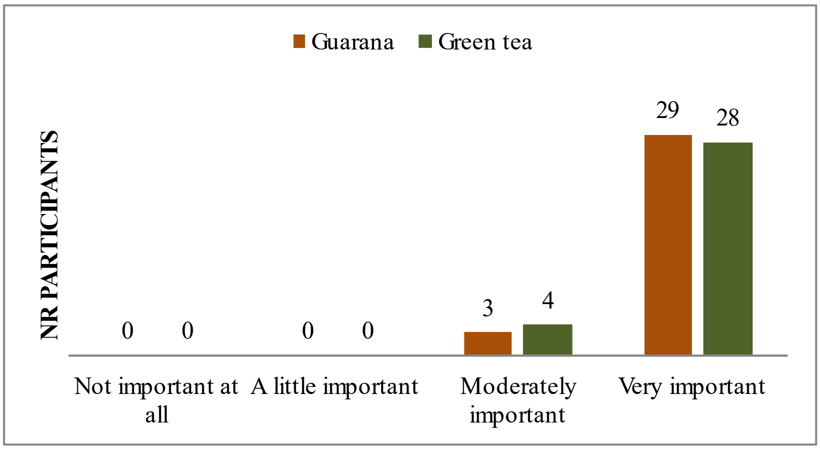

Figure S40. Perception of Green Tea/Guarana as Helping to Cope with Life

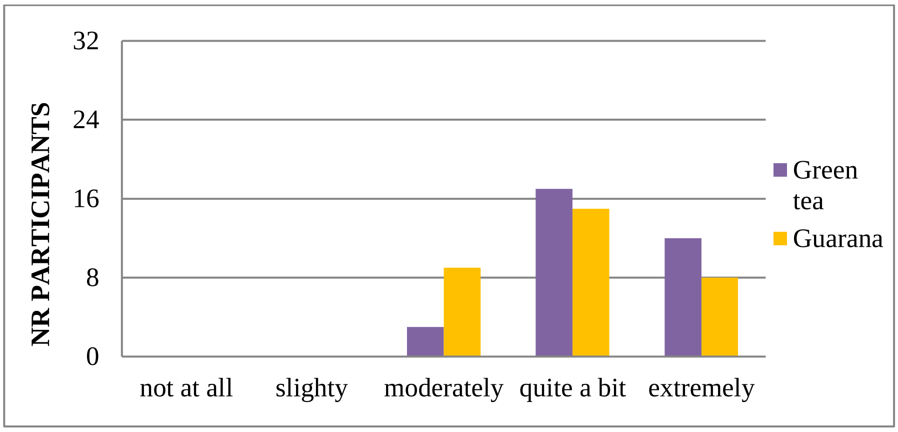

Figure S41. Perceived nutritional value of green tea/guarana

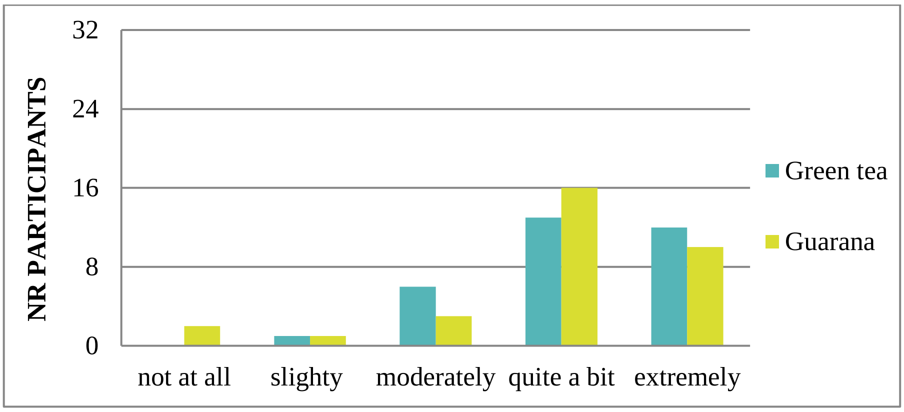

Figure S42. Willingness to Learn About and Collect Information on Green Tea/Guarana

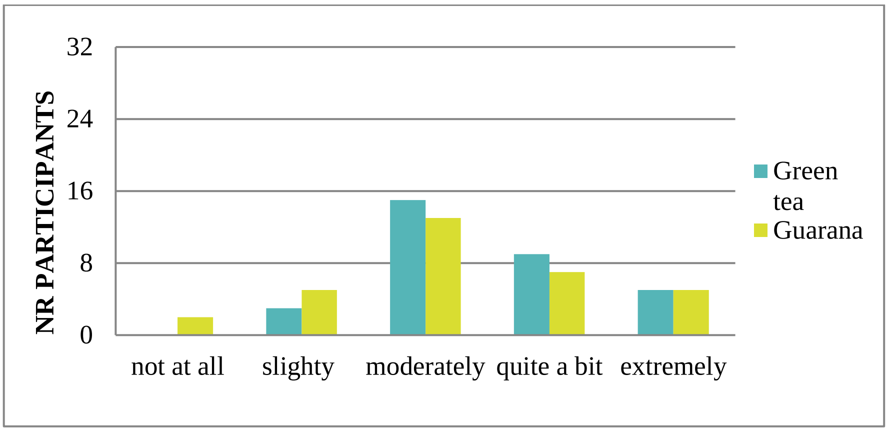

Figure S43. Willingness to Purchase Green Tea/Guarana Given Adequate Resources
